# Identification of Chilling Accumulation-Associated Genes for Litchi Flowering by Transcriptome-Based Genome-Wide Association Studies

**DOI:** 10.3389/fpls.2022.819188

**Published:** 2022-02-23

**Authors:** Xingyu Lu, Peitao Lü, Hao Liu, Houbin Chen, Xifen Pan, Pengxu Liu, Lei Feng, Silin Zhong, Biyan Zhou

**Affiliations:** ^1^Guangdong Litchi Engineering Research Center, College of Horticulture, South China Agricultural University, Guangzhou, China; ^2^College of Life and Health Science, Kaili University, Kaili, China; ^3^College of Horticulture, Fujian Agriculture and Forestry University-University of California Riverside (FAFU-UCR) Joint Center for Horticultural Biology and Metabolomics, Haixia Institute of Science and Technology, Fujian Agriculture and Forestry University, Fuzhou, China; ^4^State Key Laboratory of Agrobiotechnology, School of Life Sciences, Chinese University of Hong Kong, Shatin, Hong Kong SAR, China

**Keywords:** flowering, chilling accumulation, transcriptome, association analysis, litchi

## Abstract

Litchi is an important Sapindaceae fruit tree. Flowering in litchi is triggered by low temperatures in autumn and winter. It can be divided into early-, medium-, and late-flowering phenotypes according to the time for floral induction. Early-flowering varieties need low chilling accumulation level for floral induction, whereas the late-flowering varieties require high chilling accumulation level. In the present study, RNA-Seq of 87 accessions was performed and transcriptome-based genome-wide association studies (GWAS) was used to identify candidate genes involved in chilling accumulation underlying the time for floral induction. A total of 98,155 high-quality single-nucleotide polymorphism (SNP) sites were obtained. A total of 1,411 significantly associated SNPs and 1,115 associated genes (AGs) were identified, of which 31 were flowering-related, 23 were hormone synthesis-related, and 27 were hormone signal transduction-related. Association analysis between the gene expression of the AGs and the flowering phenotypic data was carried out, and differentially expressed genes (DEGs) in a temperature-controlled experiment were obtained. As a result, 15 flowering-related candidate AGs (CAGs), 13 hormone synthesis-related CAGs, and 11 hormone signal transduction-related CAGs were further screened. The expression levels of the CAGs in the early-flowering accessions were different from those in the late-flowering ones, and also between the flowering trees and non-flowering trees. In a gradient chilling treatment, flowering rates of the trees and the CAGs expression were affected by the treatment. Our present work for the first time provided candidate genes for genetic regulation of flowering in litchi using transcriptome-based GWAS.

## Introduction

Litchi is an important Sapindaceae fruit tree. Flowering in litchi is triggered by low temperatures in autumn and winter (Menzel and Simpson, 1988; Chen and Huang, 2005; Zhou et al., 2014). It is widely grown in southeast Asia within the latitude of 17°–26°N, and is commercially cultivated in South Africa and Australia within the latitude of 17°–32°S. These areas are normally chill in winter without freezing temperatures, in which chilling accumulation is enough for litchi floral induction. However, during the past 20 years, insufficient winter chilling attributed to global warming that has been occurring frequently. An extreme case happened in the winter of 2018–2019, in which the monthly average temperature was 17.8°C from November to January, resulting in poor flowering and low yield in 2019 (Su et al., 2020). Hence, it is important to understand the genetics of litchi flowering in relation to chilling so as to find ways for its regulation.

As the origin center of litchi, China is rich in litchi resources with diverse traits. One of the most important traits of these resources is the time for the accomplishment of floral induction, which is related to the chilling requirement. Up till now, all the litchi accessions found are evergreen fruit trees. They are grown in areas with chill winter without freezing temperatures. Unlike the deciduous fruit trees such as almond, whose leaves abscise and floral buds are at the endodormancy stage in winter (Prudencio et al., 2018, 2021), litchi floral buds are induced in winter, and winter chilling is indispensable for floral induction (Menzel and Simpson, 1988; Chen and Huang, 2005; Zhou et al., 2014). In the model plant Arabidopsis, CONSTANS (CO) is regarded as a direct activator of *FLOWERING LOCUS T* (*FT*), and the FT protein is a long-seeking florigen that migrated from leaves to the shoot apical meristem (SAM) to promote floral initiation (Corbesier et al., 2007; Yang et al., 2007). Interestingly, litchi trees grown under high-temperature conditions with only a few leaves treated with low temperature could still produce flowers (Zhang et al., 2017), suggesting the chilled leaves produced signals and migrated from leaves to the SAM to induce floral transition. Litchi resources can be divided into early-, medium-, and late-flowering groups according to the time for floral induction and blossom. The early-flowering group can undergo floral induction early, just at the very beginning of winter, suggesting that it needs a low chilling accumulation level, while the late-flowering group has to be subjected to a long period of winter chilling, suggesting that it needs a high chilling accumulation level. We have recorded the time for floral initiation of the collected accessions in our litchi germplasm garden and investigated the chilling accumulation requirement for floral induction of the accessions as basic information for years.

In the present study, we selected 87 accessions from our litchi germplasm garden and recorded the time for floral initiation, quantified the time and the chilling accumulation into quantitative traits as the flowering phenotype data. We then performed RNA-Seq of the accessions and collected population single-nucleotide polymorphism (SNP) data for transcriptome-based GWAS to identify associated genes (AGs). We also performed RNA-Seq of leaves in flowering and non-flowering trees. Based on this conjoint analysis of RNA-Seq, we narrowed the AGs and identified candidate AGs (CAGs). At last, a gradient chilling treatment of the litchi trees was carried out and the expressions of the flowering-related CAGs were further determined by quantitative real-time RT-PCR (qRT-PCR) to identify crucial genes of flowering regulation underlying chilling accumulation. Our present work for the first time provided CAGs by transcriptome-based GWAS in litchi. These genes may be applied for genetic regulation of flowering, and for bypassing or partly bypassing chilling underlying climate change and global warming.

## Materials and Methods

### Plant Material and Experimental Procedures

For the transcriptome-based GWAS, 87 litchi accessions (cultivars or lines) were selected from the germplasm resource garden in South China Agricultural University, Guangzhou, China (latitude 23°9040″ N, longitude 113°21018″ E). The origin, the pedigree, and the flowering property of the accessions are shown in Supplementary Table 1. Leaves of the accessions were collected in early December when temperature was low enough for floral induction. Twenty mature leaves collected from four directions of the terminal shoots from each accession were pooled together as one sample. The samples were immediately frozen in liquid nitrogen and stored at −80°C for RNA extraction.

To compare the gene expression pattern of the flowering and non-flowering litchi trees, 20 5-year-old air-laying potted trees (*Litchi chinensis* cv. Nuomici, 1–1.5 m height) with similar phenological stages were used for controlled temperature treatment. All the trees were grown in 30-L pots with loam, mushroom cinder, and coconut chaff (v: v: v, 3:1:1). When the terminal shoots matured, 10 trees as the non-flowering control (H, high-temperature) were transferred to a growth chamber at 25/20°C (day/night temperature, 12 h day and 12 h night) with a relative humidity of 75–85% and natural light. The remaining 10 trees, as the flowering treatment (L, low-temperature), were transferred to a growth chamber at 15/8°C with the same light and humidity conditions. The trees were rewarmed to 25/20°C after 60 days of chilling. Leaves of the L- and H-treated trees at the time points of 3, 30, 60, and 75 days (floral initiation) were sampled, and leaves of the terminal shoots from 3 to 4 trees were pooled together as a replicate (each time point with three biological replicates). For the RNA-Seq data confirmation, litchi potted trees were grown in chambers with similar temperature and light conditions. Leaves of the L- and H-treated trees at the time points of 0, 30, 60, and 75 days (floral initiation) were sampled for analysis. The samples were immediately frozen in liquid nitrogen and stored at −80°C for RNA extraction and RNA-Seq.

To further confirm the chilling accumulation-related candidate genes, the medium-late-flowering ‘Guiwei’ litchi trees were selected. A total of 15 5-year-old air-laying potted trees with similar phenological stages were grown in a growth chamber at 10°C for floral induction. The trees were evenly divided into three groups and transferred to a growth chamber at 22°C after 5 weeks (Treatment 1), 7 weeks (Treatment 2), and 9 weeks (treatment 3, sufficient chilling accumulation), respectively. Leaves of the trees at the time points of 0 day (S1), 21 days (S2), and 35 days (S3) for Treatment 1, S1, S2, S3, and 49 days (S4) for Treatment 2, S1, S2, S3, S4, and 63 days (S5) for Treatment 3 were sampled. All the leaf samples were immediately frozen in liquid nitrogen and stored at −80°C for RNA extraction.

### Data Processing of Flowering Phenotype of the Accessions

For trait surveys, we recorded the date of the emergence of panicle primordia visible as “whitish millet.” We also recorded the temperatures every 30 min from 1 September 2014 to 30 February 2015 using a temperature and humidity recorder (ZDR-M20). To quantify the specific flowering time into quantitative traits, we used three sets of data to describe the flowering phenotypes. Symbol A, flowering phenotype based on the time of panicle primordium (“whitish millet”) emergence, included eight groups. Groups 1–2, 3, 4–5, 6, 7–8 indicated the early-, the early-medium-, the medium-, the medium-late, and the late flowering accessions, respectively. Symbol B indicated flowering phenotype based on the days required for floral induction (from 1 September 2014 to the date when the “whitish millet” appeared). Symbol C indicated flowering phenotype based on chilling accumulation denoted by degree hours according to the method described by Chen et al. (2016), as the sum of the temperatures lower than 20°C from 1 September 2014 to the date when the “whitish millet” appeared.

### RNA Isolation, Library Construction, and Data Preprocessing

We chose the plant total RNA isolation kit (polysaccharides and polyphenolics-rich) (Huayueyang, Beijing, China) to extract total RNA from the litchi samples. A total of 5 μg of total RNA with an appropriate amount of Oligo-dT25 beads (Invitrogen, Carlsbad, CA, United States) was used for RNA-Seq library construction. First, the enriched mRNA was fragmented into short fragments and reverse transcribed into cDNA by random primers. Next, second-strand cDNA was synthesized using DNA polymerase I, RNase H, and dNTPs. The cDNA fragments were then purified by Agencourt AMPure XP (Beckman Coulter, Pasadena, CA, United States). The purified cDNA fragments were end-repaired, modified with poly (A) tails, and ligated to Illumina sequencing adapters. At last, the size-selected fragments were amplified and purified.

Libraries of 87 litchi accessions and 24 ‘Nuomici’ leaf samples were sequenced with Illumina HiSeq™ 2500 (BGI Co. Ltd., Shenzhen, China). The full RNA-Seq data have been submitted to the Sequence Read Archive (SRA) of the NCBI under accession PRJNA766599 and PRJNA766549.^1^ Adaptors, low-quality sequences, and rRNA sequences were removed from raw reads. Then, the cleaned reads were mapped to the litchi genome^2^ by TopHat2 (version 2.0.3.12). The eXpress (v1.5.1^3^) was used to calculate the read counts and fragments per kilobase of transcript per million mapped reads (FPKM) of each gene.

### Genotype Analysis

Genome Analysis ToolKit, 4.0.6.0 (GATK) was used for SNP-calling (Van der Auwera et al., 2013). SNPs with single sample sequencing depth ≥3, missing rate less than 0.2, and the minor allele frequency (MAF, the frequency of occurrence of uncommon alleles in the population) more than 5% were identified as the high-quality filtered SNPs. ANNOVAR (Wang et al., 2010) (version 77) was applied for SNP/InDel annotation. The sequence difference matrix between individuals was obtained from the population SNP data, and a phylogenetic tree was constructed by the neighbor-joining method in the software Treebest (version 1.9.2) and MEGA 6.0.^4^ The admixture model-based software Admixture (version 1.3.0) (Alexander et al., 2009) was used to estimate population structure and generate the Q matrix. The software SPAGeDi (Hardy and Vekemans, 2002) (version 1.5^5^) and the method described by Loiselle et al. (1995) were used to obtain the kinship matrix (K matrix).

### Association Analysis Between Flowering Phenotypic and Population Single-Nucleotide Polymorphism Data

The association analysis software TASSEL (Bradbury et al., 2007) (version 3.0) was applied for the association analysis of flowering phenotypic and population SNP data, and four models were constructed: the general linear model (GLM model); the mixed linear model with Q matrix as covariate (Q model); mixed linear model with K matrix as covariate (K model), and mixed linear model with Q matrix and K matrix as covariates (Q + K model). The value of column “Symbol A,” “Symbol B” in Supplementary Table 1, and the natural logarithm of the value of column “Symbol C” in Supplementary Table 1 were used for association analysis. Q–Q plot was used to compare the distribution of the observed (actual) *p*-value and expected (theoretical) *p*-value of the models. Then, the Bonferroni correction was applied to determine the threshold of significant level (0.05/markers number). The quantitative trait loci (QTLs) were defined as the most strongly associated SNP (lead SNP, *p*-value < 1.00E-05) loci, and the genes that contained those QTLs were defined as AGs.

### Association Analysis Between Gene Expression and the Flowering Phenotypic Data

The relationship between gene expression level of the screened chilling accumulation related AGs (flowering, hormone synthesis, hormone signal induction-related AGs) and the flowering phenotypic data (Symbol C in Supplementary Table 1) was calculated by linear regression using R.^6^ For each gene, FPKM value was regressed as the dependent variable and chilling accumulation as the independent variable, and *R*^2^ and significance values were calculated. Differentially expressed genes (DEGs) in the controlled temperature treatment were determined by using the OmicShare tools (edgeR^7^), and significant DEGs were restricted with FDR ≤0.05 and the absolute value of fold-change ≥ 1.5. Pathway enrichment analysis of the DEGs were performed using the OmicShare tools.^8^ The expression of finally selected CAGs (*p*-value < 1.00E-05) was presented as a heat map diagram by integrative toolkit TBtools (Chen et al., 2020) (v1.075).

### Quantitative Real-Time PCR Analysis

Total RNA was extracted by RNAprep Pure Plant Kit (Polysaccharides & Polyphenolics-rich) (Tiangen Biotech, China). A total of 1 μg total RNA was used for the synthesis of first-strand cDNA by the TransScript One-Step gDNA removal and cDNA synthesis SuperMix Kit (Transgen Biotech, China). Primers F/R for qRT-PCR were designed by Primer 5.0 software and synthesized at Sangon Co. Ltd. (Shanghai). The litchi homolog β-*actin* (NCBI GenBank accession number: HQ588865.1) was used as the reference gene. All the primers are shown in Supplementary Table 2. qRT-PCR was performed on a CFX96 real-time PCR machine (Bio-Rad, United States) with run as follows: 95°C for 10 min, followed by 40 cycles of 95°C for 15 s, 60°C for 30 s, and 72°C for 30 s. A 2 × RealStar Green Power Mixture (GenStar BioSolutions, China) was used in the qRT-PCR reactions, and each analysis was performed in two technical replicates and three biological replicates. The transcript quantification of the genes was performed in relation to β-*actin* and calculated using 2^–ΔΔ*CT*^ method (Livak and Schmittgen, 2001). Expressions of candidate genes were presented as a heat map diagram using pheatmap package (see text footnote 6).

## Results

### Flowering Phenotype of the Litchi Accessions

Traits associated with flowering phenotype of the 87 accessions were presented by “Symbol A,” “Symbol B,” and “Symbol C” (Supplementary Table 1). The accessions were subdivided into eight subcategories based on the time of panicle primordium emergence. Category 1–2 including 10 accessions were defined as early-flowering group. Category 3 comprising 6 accessions was the early-medium flowering group, while categories 4–5, including 11 accessions, were the medium-flowering group. Category 6 comprising 17 accessions was the medium-late-flowering group. The last group, categories 7–8 including 43 accessions were defined as the late-flowering one (“Symbol A” in Supplementary Table 1). “Symbol B” indicated the days required for floral induction. The time required for floral induction was quite different. For example, the time for the early-flowering ‘Sanyuehong’ was 54 days, while that of ‘Dongliuhao’ was 169 days. “Symbol C” indicated the chilling accumulation for floral induction. The chilling accumulation of the accessions was multiple, that in the early-flowering ‘Sanyuehong’ was about 600 times lower than in ‘Dongliuyihao’. In general, these symbols indicated a multiplicity of flowering time and chilling accumulation levels in the accessions.

### Throughput and Quality of RNA-Seq Libraries of the Litchi Accessions

We constructed RNA-Seq libraries of the 87 litchi accessions and performed transcriptome sequencing. As shown in Supplementary Table 3, 1.97 × 10^8^–4.15 × 10^9^ raw data were generated, and 1.10 × 10^6^–2.62 × 10^6^ clean reads were obtained from the libraries, with clean read ratios of 64.8–97.2%. We then mapped the clean reads to the litchi genome data, and the alignment rates were 58.1–77.8%.

### Cluster Analysis of Litchi Accessions Based on Population Single-Nucleotide Polymorphism Data

**FIGURE 1:**
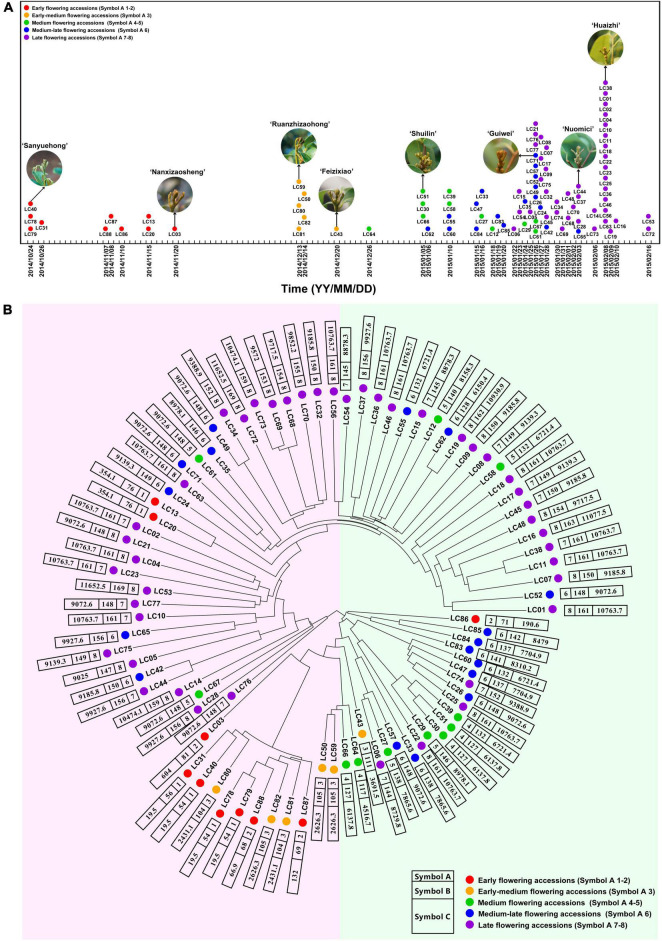
Schematic diagram of flowering time and phylogenetic tree of litchi accessions based on SNP data. **(A)** Schematic diagram of flowering time of the 87 litchi accessions. **(B)** Phylogenetic tree of the accessions based on SNP data. Symbol A, flowering phenotype based on the time of panicle primordium (“whitish millet”) emergence. Symbol 1–2, 3, 4–5, 6, 7–8 indicate early, early-medium, medium, medium-late, and late flowering accessions, respectively, and are signed by red, orange, green, blue, and purple dots. Symbol B, flowering phenotype based on days required for floral induction. Symbol C, flowering phenotype based on chilling accumulation. Detailed information of the accessions is listed in Supplementary Table 1.

A total of 98,155 high-quality SNP sites were obtained (Supplementary Table 4), and the SNP data of the population were used to obtain a sequence difference matrix among individuals, and a phylogenetic tree based on the neighbor-joining method was constructed. As shown in Figure 1, the early-flowering accessions were clustered together, so as the medium- and late-flowering ones. The results suggested that the accessions with similar flowering time possessed similar genetic background. Interestingly, ‘Khom’ (Lc13) and ‘Kaleka’ (Lc20), introduced from Thailand, have early flowering phenotype in southern China. However, their genetic backgrounds are closer to the medium-late or late-flowering accessions.

### Association Analysis Between Flowering Phenotypic Traits and Population Single-Nucleotide Polymorphism Data

The correlation trend lines of the actual *p*-values and theoretical *p*-values obtained by Q + K model based on time of panicle primordium emergence (“Symbol A”) and the days required for floral induction (“Symbol B”) were closer to the expected line (linear, *y* = *x*). However, there is almost no useful information in relation to flowering. Hence, the Q + K model with the natural logarithm of the value of chilling accumulation (“Symbol C”) was chosen to perform the associated analysis, referred to as SNP loci analysis related to chilling accumulation (Supplementary Figure 1). As a result, we identified 1,411 lead SNPs corresponding to 1,115 AGs (Supplementary Table 5).

### Screening of Candidate Associated Genes in Relation to Chilling Accumulation for Floral Induction

**FIGURE 2:**
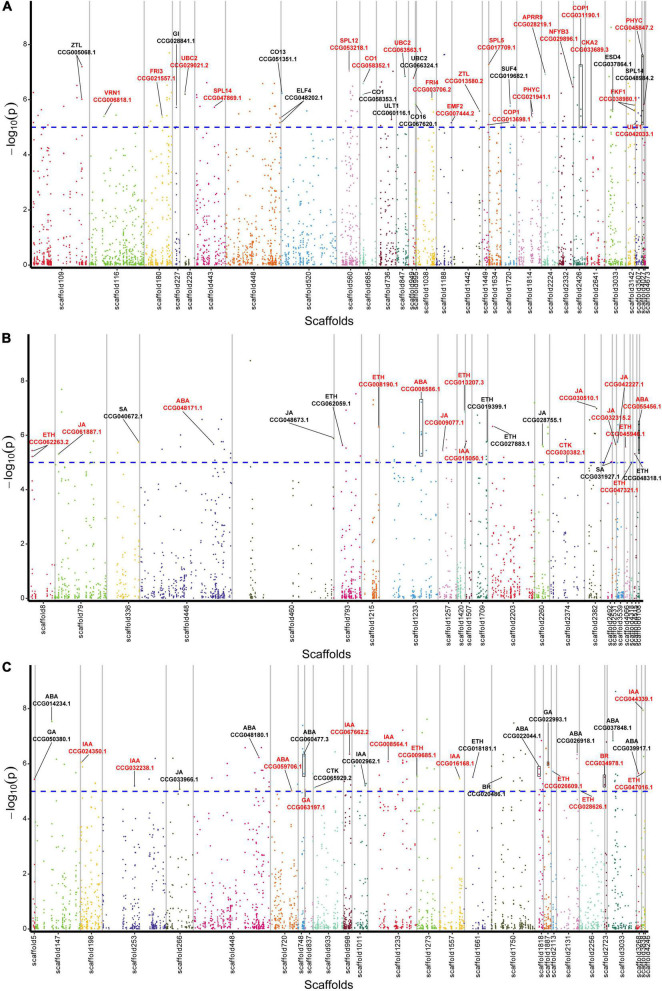
Manhattan plot displaying the location of lead SNPs corresponding associated genes. **(A)** Flowering-related AGs. **(B)** Plant hormone biosynthesis-related AGs. **(C)** Plant hormone signal transduction-related AGs. The AGs are indicated with black lines, and the CAGs are marked by red color. Horizontal dashed line indicates significance threshold of log_10_ (*p*-values) = 5.

From the 1,115 AGs, we detected 42 SNPs corresponding with 31 AGs in relation to flowering regulation (Figure 2A and Supplementary Table 6). A total of 16 genes were found to be related to photoperiod, circadian clock, and light signaling pathway, including *CO* (CCG058352.1, CCG058353.1, CCG051351.1, and CCG067620.1), *GI* (CCG028841.1), *FKF1* (CCG038980.1), *ELF4* (CCG048202.1), *ZTL* (CCG005068.1 and CCG013580.2), *COP1* (CCG013698.1 and CCG031190.1), *NFYB3* (CCG029896.1), *PHYTOCHROMO C* (CCG045847.2 and CCG021941.1), *CKA2* (CCG033689.3), and *APRR9* (CCG028219.1). Five genes were related to the vernalization pathway, including *FRI3* (CCG021557.1), *FRI4* (CCG003706.2), *SUF4* (CCG019682.1), *VRN1* (CCG006818.1), and *EMF2* (CCG007444.2). Four aging pathway-related genes *SPL5* (CCG017709.1), *SPL12* (CCG053218.1), and *SPL14* (CCG047869.1 and CCG048984.2), and other six genes *ULT1* (CCG042033.1 and CCG060116.1), *UBC2* (CCG029021.2, CCG063563.1, and CCG066324.1), and *ESD4* (CCG037864.1) were identified.

Among the 1,115 AGs, 34 SNPs corresponding with 23 hormone synthesis-related AGs were identified, including 1 auxin (IAA), 1 cytokinin (CTK), 3 abscisic acid (ABA), 9 ethylene (ETH), 7 jasmonic acid (JA), and 2 salicylic acid (SA) synthesis related genes (Figure 2B and Supplementary Table 6).

A total of 27 plant hormone signal transduction AGs corresponding with 32 SNPs were identified, including 7 IAA, 1 CTK, 3 gibberellin (GA), 8 ABA, 5 ETH, 2 brassinosteroid (BR), and 1 JA signal transduction related genes (Figure 2C and Supplementary Table 6).

To further screen CAGs, we performed association analysis between gene expression levels of the AGs and the flowering phenotypic data. As a result, we identified 20 flowering-related CAGs out of those of the 31 AGs, 15 hormone biosynthesis-related CAGs out of the 23 AGs, and 13 hormone signal transduction-related CAGs out of the 27 AGs. Totally, 59% of the AGs were further screened (Figure 2 and Supplementary Table 7).

### Digital Transcriptomic Analysis of the Controlled Temperature-Treated Trees

To verify whether the CAGs are involved in flowering, we performed controlled temperature treatment with the ‘Nuomici’ litchi trees, a widely cultivated late-flowering variety. The result showed that the trees treated with low temperature (L) flowered 100%, while those treated with relatively high temperature (H) did not flower. We then performed RNA-Seq of the 24 leaf samples from the flowering and non-flowering treated trees. As shown in Supplementary Table 8, the clean reads of the samples were 3.69 × 10^6^–9.75 × 10^6^, and the alignment rates were all above 82%. We conducted a pairwise comparison of low- and high-temperature treatment at the same time point to identify DEGs using edgeR, and 17,727 DEGs were obtained, of which 8,782 DEGs were from the 3-day time point, 7,927 DEGs were from the 30-day time point, 9,036 DEGs were from the 60-day time point, and 4,180 DEGs were from the 75-day time point (Supplementary Table 9). The KEGG pathway enrichment analysis of the DEGs showed that the plant–pathogen interaction (ko04626), plant hormone signal transduction (ko04075), starch and sucrose metabolism (ko00500), RNA transport (ko03013), spliceosome (ko03040), RNA degradation (ko03018), carbon metabolism (ko01200), ribosome biogenesis in eukaryotes (ko03008), amino sugar and nucleotide sugar metabolism (ko00520), and protein processing in endoplasmic reticulum (ko04141) pathways were significantly enriched (Supplementary Table 9).

### Expression Profiles of the Candidate Associated Genes in 87 Accessions and in Trees With Temperature-Controlled Treatment

**FIGURE 3:**
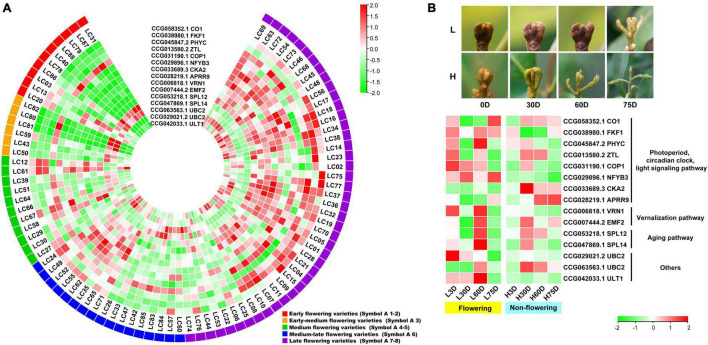
Heat map diagram of the expression of the flowering-related CAGs. **(A)** Heat map of the expression levels of the flowering-related CAGs in the 87 litchi accessions. Symbol 1–2, 3, 4–5, 6, 7–8 indicate the early (red), the early-medium (orange), the medium (green), the medium-late (blue), and the late flowering accessions (purple), respectively. **(B)** Morphology of the buds and heat map diagram of the expression levels of the CAGs of the ‘Nuomici’ litchi trees under high temperature (25/20°C, day/night temperature, 12 h day and 12 h night) and low temperature (15/8°C, day/night temperature, 12 h day and 12 h night) conditions. L3D, L30D, L60D, and L75D indicate 3, 30, 60, and 75 days of the low-temperature treatment, respectively. H3D, H30D, H60D, and H75D indicate 3, 30, 60, and 75 days of high-temperature treatment, respectively. FPKM values were normalized to *Z*-score.

An overlap of 797 genes was found from the CAGs of the transcriptome-based GWAS and DEGs in the controlled temperature experiment, during which 15 were flowering-related, 13 were hormone biosynthesis-related, and 11 were hormone signal transduction-related. Heat map diagram of the 15 flowering-related CAGs indicated *CO1* (CCG058352.1), *FKF1* (CCG038980.1), *PHYC* (CCG045847.2), *COP1* (CCG031190.1), *NFYB3* (CCG029896.1), *CKA2* (CCG033689.3), *APRR9* (CCG028219.1), *VRN1* (CCG006818.1), *EMF2* (CCG007444.2), *SPL12* (CCG053218.1), *UBC2* (CCG029021.2), *UBC2* (CCG063563.1), and *ULT1* (CCG042033.1) showed high expression levels in most of the late-flowering accessions, but low levels in most of the early-flowering and early-medium flowering accessions. *ZTL* (CCG013580.2), *SPL14* (CCG047869.1), and *UBC2* (CCG029021.2) showed low expression levels in most of the late-flowering accessions, but high expression levels in most of the early- and early-medium flowering accessions (Figure 3A). Interestingly, during the floral induction period (L3D, L30D, and L60D), expression patterns of the flowering-related CAGs in the flowering trees are contrary to those in the non-flowering ones (Figure 3B).

**FIGURE 4:**
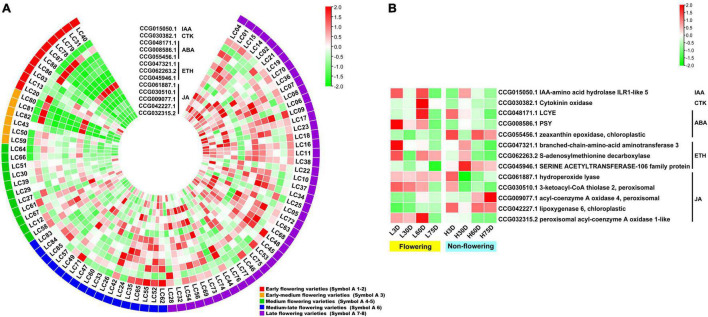
Heat map diagram of the expression of hormone biosynthesis-related CAGs. **(A)** Heat map of the expression levels of the hormone synthesis-related CAGs in the 87 litchi accessions. **(B)** Heat map diagram of the expression levels of flowering-related CAGs under low and high temperature conditions. L3D, L30D, L60D, and L75D indicate 3, 30, 60, and 75 days of the low-temperature treatment, respectively. H3D, H30D, H60D, and H75D indicate 3, 30, 60, and 75 days of the high-temperature treatment, respectively. FPKM values were normalized to *Z*-score.

For the hormone synthesis-related CAGs, as shown in Figure 4A, the expression levels of the 13 genes in the early- and the early-medium flowering accessions are different from those in the other accessions. The CAGs related to the synthesis of IAA (CCG015050.1), ABA (CCG048171.1, CCG008586.1, and CCG055456.1), ETH (CCG047321.1 and CCG062263.2), and JA (CCG061887.1, CCG030510.1, CCG009077.1, CCG042227.1, and CCG032315.2) showed low-expression levels in most of the early- and the early-medium flowering accessions, with the exception of the 2 CAGs related to synthesis of CTK (CCG030382.1) and ETH (CCG045946.1). In the controlled temperature treatment, similar to the flowering-related CAGs, the expression pattern of the hormone synthesis-related CAGs in the low temperature-treated trees are contrary to those in the high temperature treated ones during the floral induction period (Figure 4B).

**FIGURE 5:**
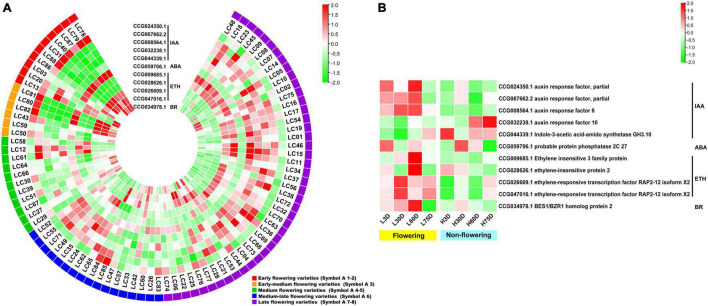
Heat map diagram of the expression of the hormone signal induction-related CAGs. **(A)** Heat map of the expression levels of the hormone signal induction-related CAGs in the 87 litchi accessions. **(B)** Heat map diagram of the expression levels of flowering-related CAGs under low and high temperature conditions. L3D, L30D, L60D, and L75D indicate 3, 30, 60, and 75 days of the low-temperature treatment, respectively. H3D, H30D, H60D, and H75D indicate 3, 30, 60, and 75 days of the high-temperature treatment, respectively. FPKM values were normalized to *Z*-score.

For the hormone signal transduction-related CAGs, the expression levels of the 11 CAGs in the early- and early-medium flowering accessions are quite different from the other accessions. The CAGs related to hormone signal transduction of IAA (CCG024350.1), ABA (CCG059706.1), ETH (CCG026609.1 and CCG047016.1), and BR (CCG034978.1) showed high expression levels in most of the early- and early-medium flowering accessions, whereas those related to hormone signal transduction of IAA (CCG067662.2, CCG008564.1, CCG032238.1, and CCG044339.1) and ETH (CCG009685.1 and CCG028626.1) showed low expression levels (Figure 5A). In the controlled temperature treatment, the CAGs in the low temperature-treated trees are quite different from those in the high temperature treated ones. Most of the CAGs showed opposite expression levels between the low and the high temperature-treated trees. Meanwhile, except for genes related to the signal transduction of IAA (CCG032238.1 and CCG044339.1), most of the CAGs showed high expression levels during the floral induction period and decreased expression levels at the floral initiation stage. Also, they showed relatively low expression levels in the non-flowering trees (Figure 5B).

**FIGURE 6:**
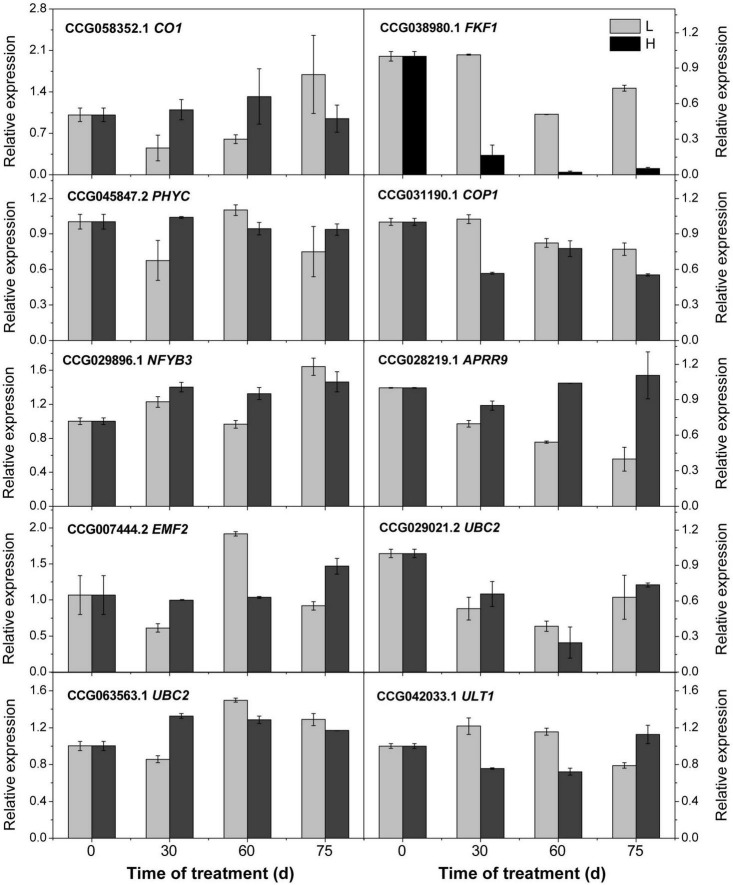
Relative expression of the candidate genes revealed by quantitative real-time PCR. L and H indicate the low-temperature and high-temperature treatment, respectively. The data are the means of three biological replicates, and the bars represent SEs. The litchi homolog β-*actin* was used as a reference gene.

To confirm the accuracy of the transcriptome analysis results, we randomly selected 10 genes for qRT-PCR confirmation, and the results suggesting the transcriptome analysis by RNA-Seq is reliable (Figure 6).

### Expression of the Candidate Associated Genes Under Gradient Chilling Conditions

**FIGURE 7:**
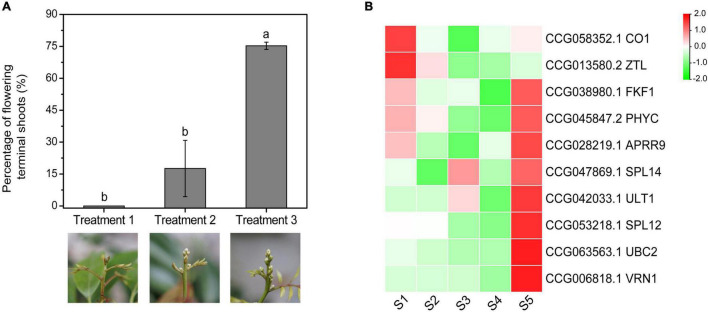
Effects of gradient chilling treatments on flowering **(A)** and CAG expression **(B)** of litchi trees. ‘Guiwei’ potted trees were grown in a growth chamber at 10°C for floral induction. The trees were transferred to a growth chamber at 22°C after 5, 7, and 9 weeks, respectively. Different letters indicate significant differences at *p* < 0.05 among the treatments according to Tukey’s multiple range test. S1, S2, S3, S4, and S5 indicate the time points of 0, 21, 35, 49, and 63 days of chilling.

To investigate the expression patterns of the CAGs under gradient chilling conditions, the medium-late-flowering accession, ‘Guiwei’ litchi trees were grown in a growth chamber at 10°C for 5 (Treatment 1), 7 (Treatment 2), and 9 (Treatment 3) weeks. A gradient increase in the flowering rate of the treatments was found. Trees of Treatment 1 did not flower, while those of Treatment 2 and 3 flowered with a percentage of flowering terminal shoots of 17.60 and 75.24%, respectively (Figure 7A).

Expressions of *CO1* and *ZTL* showed overall decreasing trends, whereas those of the *SPL12*, *UBC2*, *VRN1*, *SPL14*, and *ULT1* showed overall rising trends. *FKF1*, *PHYC*, and *APRR9* remained at low levels before S4, and increased sharply at S5, a time point for enough chilling accumulation for floral induction. In general, except for *CO1* and *ZTL*, expression levels of the flowering-related CAGs increased sharply at the time point when the trees obtained sufficient chilling accumulation for floral induction (Figure 7B).

### Hypothesis of the Chilling Accumulation Related Genes in the Regulation of Flowering Time in Litchi

**FIGURE 8:**
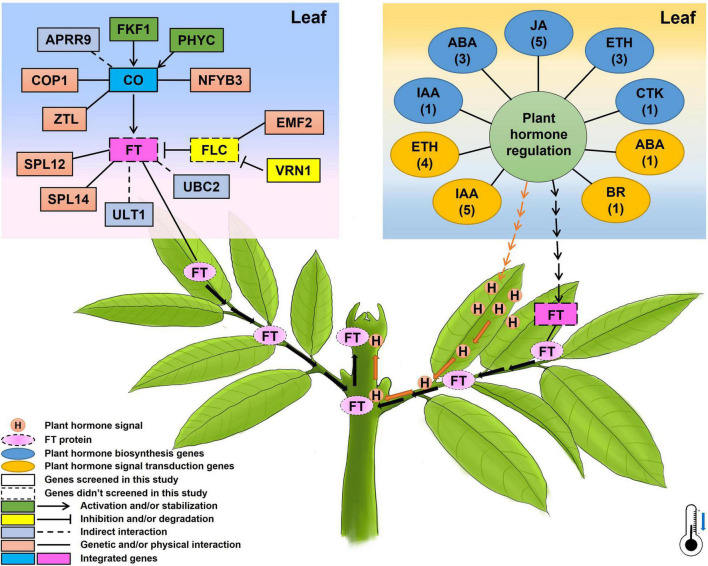
A proposed diagram showing the possible genetic pathways regulating flowering time/floral transition underlying chilling accumulation in litchi. The numbers below the names of plant hormones in the oval boxes represent the members of the identified CAGs, and the thermometer with a blue arrow indicates the low temperature required for litchi flowering in winter.

Based on the above-presented results, a potential mechanism of flowering time underlying chilling accumulation in litchi was proposed and presented in Figure 8. In leaves, under winter chilling or controlled chilling conditions, flowering-related genes such as the *FKF1* and *PHYC* promote *CO* accumulation, and the *APRR9*, *COP1*, *ZTL*, and *NFYB3* have a direct or indirect interaction with *CO*. CO activates the transcription of *FT*. In the phloem system, the FT protein acts as florigen, migrating from leaves to the SAMs to induce floral transition. Other flowering-related genes such as *SPL12*, *SPL14*, *ULT1*, and *UBC2* directly or indirectly promote *FT* expression. *VRN1* and *EMF2* may affect the expression of the *FLC* whose protein acts as a repressor to inhibit the expression of *FT*. Also, chilling may change the hormonal regulation system by affecting the expression of the plant hormone biosynthesis or signal transduction-related genes. The changes in the hormonal regulation system may on one hand produce hormone signals which can move from leaves to the apical meristems through vascular systems to promote floral transition, and on the other hand may promote the *FT* expression by several pathways. In the shoot apex, the migrated FT proteins and the hormone signals may synergistically promote floral transition.

## Discussion

Flowering is a transition from vegetative growth to reproductive growth. The complexity of this transition is created by an intricate network of signaling pathways which comprise diverse genes. Identification of these genes is important for crop production. In the past few years, genome-wide association studies (GWAS) have been widely used in rice (Huang et al., 2012), soybean (Zhou et al., 2015), maize (Tian et al., 2011), and melon (Pereira et al., 2018) for the identification of important trait-controlled genes. It has also been used to identify flowering-related genes in Arabidopsis (Aranzana et al., 2005), rice (Liu et al., 2021), maize (Maldonado et al., 2019), chrysanthemum (Chong et al., 2019), and soybean (Kim et al., 2020). Right now, no publication has reported any flowering time-related genes identified by GWAS in litchi. GWAS is useful for detecting potential targeted loci and candidate genes responsible for variations in complex traits (Bac-Molenaar et al., 2015; Sun et al., 2017). Transcriptome-based GWAS is a means of reducing sequence complexity from that represented by the entire genome (Harper et al., 2012; Galpaz et al., 2018). With the advantages of simplifying analysis and reducing cost, transcriptome-based GWAS can also analyze the variation in different individuals (Zhu et al., 2018). Hence, in the present study, we used a transcriptome-based GWAS to identify flowering time-related genes in litchi.

Litchi is a woody fruit tree with a long-life cycle as well as a long juvenile phase. Time and cost are involved to construct hybrid populations for association analysis based on flowering-related phenotype. In the present study, we used a natural group of the collected accessions in our germplasm resource garden. The accessions were from China, Thailand, South Africa, Australia, and Bangladesh with diverse flowering time. Also, they were planted at the same time in our germplasm resource garden to provide a similar growing environment and comparable flowering phenotypic data of the accessions. We constructed a phylogenetic tree, showing that most of the litchi accessions were clustered in accordance with the flowering time, suggesting that individuals with similar flowering time may have a similar genetic background. Interestingly, we found that the background of two early-flowering accessions, ‘Khom’ (Lc13) and ‘Kaleka’ (Lc20) are closer to those medium-late- or late-flowering accessions. Although they are introduced from Thailand where the ecological condition is different, they belong to the *L. chinensis*. Some mutations may happen so that they can flower under a warmer climate condition. Further study should be carried out to investigate the flowering mechanism of these interesting accessions.

For quantification of the specific floral induction time period into quantitative traits for association analysis, we used three kinds of flowering phenotypic traits. We finally identified lead SNPs using the data based on the natural logarithm of the value of degree hours (Symbol C) rather than those just based on time (Symbol A and B). Previous studies have shown that low temperature is required for litchi floral induction (Menzel and Simpson, 1988; Chen and Huang, 2005; Zhou et al., 2014). Chen et al. (2016) established a temperature model for litchi flowering and provided a base temperature of 19.32 for the sum of chilling accumulation. In our study, we used 20°C as the base temperature. We proposed that the base temperature should cover all the accessions including the early flowering accessions so as to get comparable phenotypic data. According to our previous study in the controlled temperature treatments, the early flowering accession ‘Sanyuehong’ could stably undergo floral induction in growth chambers at no more than 20°C (Liu, 2017). Hence, in the present study, we selected 20°C as the base temperature. Actually, the result showed that the chilling accumulation data were more workable for association analysis.

It is well studied that *FT* or its homolog encodes a long-seeking florigen protein that migrates from the leaves to the apical meristem to promote floral initiation (Corbesier et al., 2007; Yang et al., 2007). In the apical meristem, it interacts with the FLOWERING LOCUS D (FD) to promote floral transition and to initiate floral development through transcriptional activation of a floral meristem identity gene, *APETALA1* (*AP1*) (Abe et al., 2005). Ding et al. (2015) showed that *LcFT* expressed in leaves plays a pivotal role in litchi floral induction. As the leaves are part of the chilling sensing organs, and to produce signal for floral transition, in the present work, we focused on the chilling effects on the litchi leaves in which they produce flowering signals. We collected the leaf samples for the association and the transcriptomic analysis, and recorded the emergence time point of panicle primordia, which were visible as “whitish millets,” symbolizations of the accomplishment for floral induction. Interestingly, our research on temperature-controlled treatment indicated that *LcFT1* (CCG039962.1) and *LcFT2* (CCG035605.1) in leaves of the low temperature-treated trees were also activated by the chilling treatment, whereas no expression levels of the genes were detected in the non-flowering trees (Supplementary Table 9), suggesting that *LcFT1/2* plays important roles in flowering. It may be that the sequence of *FT* is conserved in the litchi accessions and *FT* functions as a downstream integrated gene in flowering regulation, and we did not screen the *LcFT1/2* in the transcriptome-based GWAS. Huang and Chen (2005) pointed out that apical buds were in the dormant stage, and floral initiation occurs from this stage onward when cell division becomes active. There are three types of dormancy: endodormancy, paradormancy, and ecodormancy (Lang et al., 1987). In the temperate fruit tree almond, flowering is a complex process involving chilling and heat requirements. The chilling requirements mainly affects endodormancy periods and are for breaking dormancy, and the heat requirements affect ecodormancy and are for flowering and productivity (Prudencio et al., 2018). Unlike deciduous temperate almond whose dormancy mechanism is well studied (Prudencio et al., 2019, 2021), there is less information about the evergreen litchi. Hieke et al. (2002) pointed out that the dormancy in litchi buds is not paradormancy or ecodormancy. It may be the endodormancy, and it is not induced or released by environmental cues but a purely endogenous process (Zhang et al., 2016). It might be that the chilling accumulation mainly induced the floral signals in leaves. However, how these signals affect the apical buds and promote the break out of the “whitish millets” still needs further investigation.

The GWAS is powerful for identifying candidate genes corresponding to natural variation of traits in field crops as well as in horticultural plants (Su et al., 2019; Alseekh et al., 2021; Berhe et al., 2021). A larger population size has been used for association analysis in field crops, such as rice (Huang et al., 2012), soybean (Zhou et al., 2015), and maize (Maldonado et al., 2019). GWAS has also been identified in trait-related candidate genes or SNPs in horticultural plants using populations less than 100, such as rose (Schulz et al., 2016), chestnut (Kang et al., 2019), chrysanthemums (Su et al., 2019), and bigleaf hydrangea (Wu and Alexander, 2020). In this research, we used 87 litchi accessions for the transcriptome-based GWAS and obtained 1,411 lead SNPs and 1,115 AGs. Then, we performed association analysis between gene expression and the flowering phenotypic data. Then a temperature-controlled experiment was carried out and 15 flowering-related CAGs, 13 hormone synthesis-related CAGs, and 11 hormone signal transduction-related CAGs were finally obtained. Interestingly, the expression levels of the 48 CAGs in the early and early-medium flowering accessions were quite different from the late flowering accessions (Figures 3A, 4A, 5A), and the possible reason might be laid on the different genetic back grounds as shown in the phylogenetic tree based on population SNP data (Figure 1). In this study, the lead SNPs were located on the CDS or 3′- or 5′-untranslated regions of the corresponding CAGs. The sequential difference of the CAGs between the early/early-medium flowering accessions and the late flowering accessions might affect their expression. Interestingly, most of the CAGs were highly expressed in the flowering trees and lowly expressed in the non-flowering trees just at the same time points (Figures 3B, 4B, 5B), suggesting that expression patterns of these CAGs in the flowering trees were quite different from those in the non-flowering ones. The results further confirm that the CAGs are related to flowering time/chilling accumulation.

Plant hormones are signal molecules produced in plant cells in response to environment and are expressed at extremely low concentrations, but regulate a wide range of processes, including floral transition (Davis, 2009). We have identified IAA, ABA, ETH, CTK, BR, and JA synthesis-related or signal transduction-related CAGs. Interestingly, the DEGs in the controlled-temperature treatment were enriched in the plant hormone signal transduction pathway. Gimode et al. (2020) identified *PP2C* as a candidate flowering time gene in watermelon using QTL-Seq method. Phosphatases (DBP), which exhibit protein phosphatase 2C activities, are important regulators that are involved in both the transcriptional and posttranslational regulations. It can suppress *FLC* expression and enhance the expression levels of CO and *FT* in Arabidopsis (Zhai et al., 2016). We also identified an ABA signal transduction related gene *PP2C* (CCG059706.1). Brassinosteroids (BRs) are plant steroid hormones and play important roles in plant growth and development. The BR signal transduction components BES1/BZR1 can interact with two proteins, ELF6 (early flowering 6) and its homolog REF6 (relative of early flowering 6) to regulate flowering time in Arabidopsis (Yu et al., 2008). In accordance with the Arabidopsis, litchi flowering was proven to be regulated by ABA (Cui et al., 2013) and BR (Hu et al., 2018). The identified hormone-related CAGs may provide candidate genes for genetic regulation of flowering underlying hormonal control.

We also identified flowering-related CAGs whose homologs in the model plant Arabidopsis are proved to be involved in flowering, such as photoperiod or circadian clock regulation, and vernalization. Unlike the herbaceous Arabidopsis, litchi is an evergreen fruit tree whose flowering mechanism may be different. The known flowering regulation pathways in the model plant may be different from those in litchi. It is likely that the regarded photoperiod pathway related genes whose homologs in litchi may play different roles in flowering. In this study, those kinds of genes such as *CO1*, *FKF1*, *PHYC*, *ZTL*, and *COP1* may be involved in the regulation of flowering time underlying chilling accumulation in litchi. The identified candidate gene was *VRN1* (CCG006818.1), whose homolog plays an important role in vernalization and flowering in Arabidopsis (Levy et al., 2002). Its homolog in wheat, encoding a MADS-box transcription factor, could be significantly induced in leaves by vernalizations, and acts as the central regulator of wheat vernalization-induced flowering (Xie et al., 2021). *APRR9* (CCG028219.1) plays a general and an important role in the mechanisms underlying circadian rhythms in Arabidopsis. The APRR9-ox plants flowered much earlier than wild-type plants (Matsushika et al., 2002). On the whole, the expression of the CAGs showed significant difference between the early flowering and late flowering accessions. In addition, the expression profile of the CAGs showed different trends between flowering litchi trees and the non-flowering ones. Taken together, the screened CAGs may be related with flowering in litchi. Further functional studies should be carried out to investigate the role of the identified genes in litchi flowering underlying chilling accumulation.

As the late- or medium-late- flowering accessions need high chilling accumulation levels for floral induction, we supposed that flowering of these trees might have a dose-dependence on chilling accumulation, and the CAGs expression levels may change with the increase in chilling accumulation levels. We then performed an experiment in growth chambers. The medium-late-flowering accession, ‘Guiwei’ potted trees were grown under gradient chilling conditions. In accordance with our hypothesis, the flowering rates of the trees showed an increasing trend with the increase in chilling accumulation levels. Moreover, the expression of the flowering-related CAGs such as *FKF1*, *APRR9*, *PHYC*, *SPL12*, *SPL14*, *VRN1*, *ULT1*, and *UBC2* showed a sudden increase at the time point when the trees got sufficient chilling accumulation for flowering, suggesting these CAGs might need sufficient chilling accumulation for high expression.

## Conclusion

We have performed transcriptome-based GWAS, obtained 1,411 lead SNPs and 1,115 AGs. To further screen CAGs, we performed association analysis between gene expression levels of the chilling accumulation related AGs and the flowering phenotypic data. We also performed a temperature-controlled experiment on litchi trees and explored the expression patterns of the screened CAGs in the flowering and non-flowering trees. Based on this conjoint analysis of RNA-Seq, we identified 15 flowering-related, 13 hormone biosynthesis-related, and 11 hormone signal transduction-related CAGs underlying chilling accumulation and flowering time in litchi. These genes may directly or indirectly affect the florigen or hormone signals produced in leaves that can migrate to the shoot apical apex to promote floral transition. Our present work for the first time provided CAGs by transcriptome-based GWAS in litchi. These genes may be applied for genetic regulation of flowering and for bypassing or partly bypassing chilling underlying climate change and global warming.

## Data Availability Statement

The datasets presented in this study can be found in online repositories. The names of the repository/repositories and accession number(s) can be found below: https://www.ncbi.nlm.nih.gov/, PRJNA766599; https://www.ncbi.nlm.nih.gov/, PRJNA766549.

## Author Contributions

BZ and SZ contributed to the design of the research and were contributors in writing the manuscript. XL performed sample collection, RNA isolation, library construction, association analysis, and writing the manuscript. PL carried out library construction and RNA-Seq data analysis. HL performed gene expression analysis. LF performed RNA-Seq data analysis. HC participated in the design of the research. XP carried out data analysis. PXL performed the phenotyping. All authors read and approved the manuscript.

## Conflict of Interest

The authors declare that the research was conducted in the absence of any commercial or financial relationships that could be construed as a potential conflict of interest.

## Publisher’s Note

All claims expressed in this article are solely those of the authors and do not necessarily represent those of their affiliated organizations, or those of the publisher, the editors and the reviewers. Any product that may be evaluated in this article, or claim that may be made by its manufacturer, is not guaranteed or endorsed by the publisher.

## Footnotes

1
https://www.ncbi.nlm.nih.gov/sra

2
http://121.37.229.61:82/

3
https://pachterlab.github.io/eXpress/index.html

4
http://www.megasoftware.net/

5
http://ebe.ulb.ac.be/ebe/SPAGeDi.html

6
http://www.r-project.org/

7
https://www.omicshare.com/tools/Home/Soft/diffanalysis

8
http://www.omicshare.com/tools/Home/Soft/pathwaygsea
