# Supplementary material for: Identification of Chilling Accumulation-Associated Genes for Litchi Flowering by Transcriptome-Based Genome-Wide Association Studies
Source: Front Plant Sci. 2022 Feb 23;13:819188. doi: 10.3389/fpls.2022.819188 (PMC8905319; doi:10.3389/fpls.2022.819188)
Supplement: Supplementary file 1 [file Data_Sheet_1.pdf]

Captions of Supplementary Tables and Figures

Tables:

Supplementary Table 1. Information of different flowering phenotypes in the 87 litchi accessions.

Symbol A, flowering phenotype based on the time of panicle primordium ("whitish millet") emergency, totally eight groups, symbol 1-2, 3, 4-5, 6, 7-8 indicated the early flowering accessions, the early-medium flowering accessions, the medium flowering accessions, the medium-late flowering accessions, and the late flowering accessions, respectively. Symbol B, flowering phenotype based on days required for floral induction (from September 1, 2014 to the date when the "whitish millet" appeared). Symbol C, flowering phenotype based on chilling accumulation indicated by Degree.Hours as the sum of the temperatures lower than 20°C from September 1 in 2014 to the date when the "whitish millet" appeared.

Supplementary Table 2. Primer sequences of the 12 CAGs for qRT-PCR

Supplementary Table 3. Data quality and alignment analysis of 87 litchi accessions.

Supplementary Table 4. SNP detection and filtering.

Supplementary Table 5. SNPs and AGs screened by associated analysis (with using Q + K model and the natural logarithm of the value of chilling accumulation as the flowering phenotypic data).

CHR indicates the number of scaffold; Site indicates the position of the SNP in the scaffold; P-value indicates the significance levels; R^2 indicates the percentage of phenotypic variation explained by each SNP; AGs indicate the associated genes.

Supplementary Table 6. Chilling accumulation-related SNP loci associated genes (genes involved in flowering regulation, plant hormone biosynthesis and plant hormone signal transduction).

CHR indicates the number of scaffold; Site indicates the position of the SNP in the scaffold.

Supplementary Table 7. Results of association analysis between the expression of chilling accumulation related AGs and the flowering phenotypic data.

P-value indicates the significance levels and R^2 indicates the correlation coefficient. The CAGs (candidate associated genes) were marked by red.

Supplementary Table 8. Data quality and alignment analysis of different temperature treated 'Nuomici' litchi samples.

'Nuomici' litchi trees under high temperature (25 °C/20 °C, day/night temperature, 12 h day and 12 h night) and low temperature (15 °C/8 °C, day/night temperature, 12 h

day and 12 h night) conditions. L3D, L30D, L60D, and L75D indicate 3, 30, 60, 75 days of the low-temperature treatment, respectively. H3D, H30D, H60D, and H75D indicate 3, 30, 60, 75 days of high-temperature treatment, respectively. -1, -2 and -3 represent the replications.

Supplementary Table 9. Digital transcriptomic analysis of different temperature treated 'Nuomici' litchi.

Figure:

Supplementary Figure 1. QQ plots of flowering phenotypic data and population SNP data.

(A) Four models based on the value of column 'Symbol A' in Supplementary Table 1 as the flowering phenotypic data; **(B)** Four models based on the value of column 'Symbol B' in Supplementary Table 1 as the flowering phenotypic data; **(C)** Four models based on the natural logarithm of the value of column 'Symbol C' in Supplementary Table 1 as the flowering phenotypic data. The QQ plot shows comparison of the distribution of expected (theoretical) $\log_{10}$ p-values (X-axis) and the distribution of observed (actual) $\log_{10} p$-values (Y-axis).
