# Supplementary figures and images for: Identification of Chilling Accumulation-Associated Genes for Litchi Flowering by Transcriptome-Based Genome-Wide Association Studies
Source: Front Plant Sci. 2022 Feb 23;13:819188. doi: 10.3389/fpls.2022.819188 (PMC8905319; doi:10.3389/fpls.2022.819188)

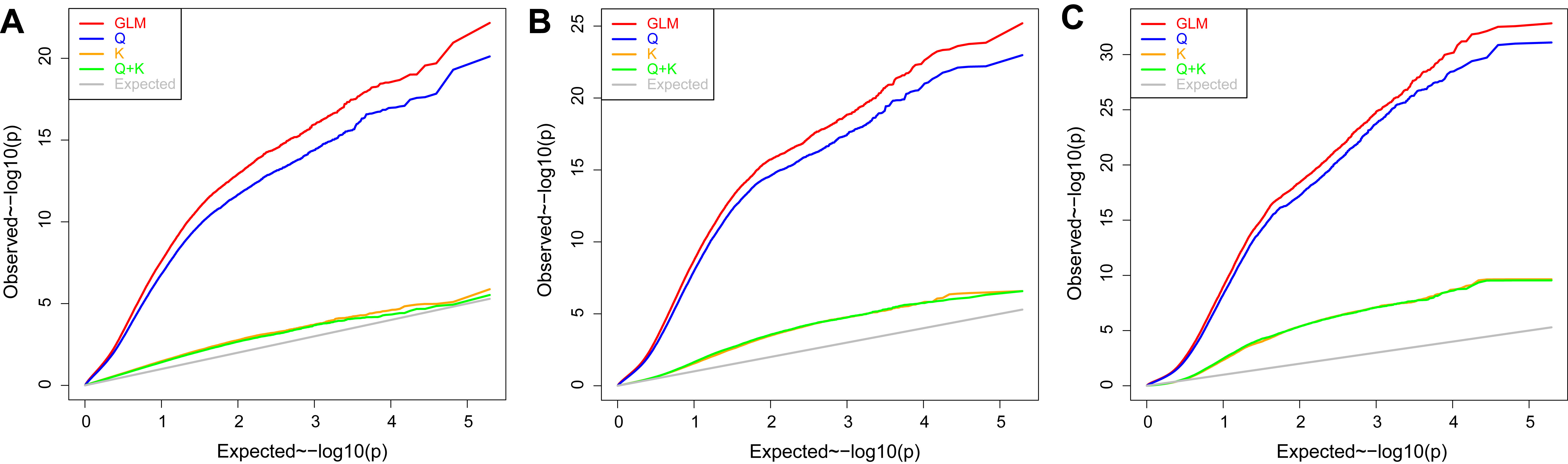

Supplement: Supplementary file 2 [file Image_1.jpg]
